# Implementing buprenorphine for opioid use disorder in veterans health administration primary care: a qualitative analysis

**DOI:** 10.1186/s13722-025-00568-9

**Published:** 2025-04-30

**Authors:** Aline Lott, Anissa N. Danner, Carol A. Malte, Hope A. Salameh, Diana Bachowski, Adam J. Gordon, Hildi J. Hagedorn, Madeline C. Frost, Emily C. Williams, Andrew J. Saxon, Ryan S. Trim, Eric J. Hawkins

**Affiliations:** 1 Health Systems Research (HSR) Center of Innovation for Veteran-Centered and Value-Driven Care, Veterans Affairs (VA) Puget Sound Health Care System, 1660 S. Columbian Way, Seattle, WA 98108 USA; 2 Center of Excellence in Substance Addiction Treatment and Education, VA Puget Sound Health Care System, Seattle, WA USA; 3 Center for Care Delivery & Outcomes Research, Health Services Research & Development, Minneapolis VA Health Care System, Minneapolis, MN USA; 4 Informatics, Decision-Enhancement, and Analytic Sciences Center, Health Systems Research, VA Salt Lake City Health Care System, Salt Lake City, UT USA; 5 Program for Addiction Research, Clinical Care, Knowledge, and Advocacy, Department of Internal Medicine, University of Utah School of Medicine, Salt Lake City, UT USA; 6 Department of Psychiatry, University of Minnesota, Minneapolis, MN USA; 7 Department of Health Systems and Population Health, University of Washington School of Public Health, Seattle, WA USA; 8 Department of Psychiatry and Behavioral Sciences, University of Washington School of Medicine, Seattle, WA USA; 9 Center of Excellence in Substance Addiction Treatment and Education, Corporal Michael J. Crescenz Philadelphia VA Medical Center, Philadelphia, PA USA

**Keywords:** Buprenorphine, Opioid use disorder, OUD care, Primary care

## Abstract

**Background:**

Medications for opioid use disorder are evidence-based, guideline-recommended treatments. While buprenorphine can be prescribed in nonspecialized office-based settings, it is underutilized. Using a multifaceted implementation initiative, the Veterans Health Administration (VHA) sought to expand access to buprenorphine in nonspecialized office-based settings, including primary care clinics. The purpose of this qualitative evaluation was to assess and describe primary care clinicians’ perspectives on delivering buprenorphine care during the first year of the initiative.

**Methods:**

Using a snowball sampling approach, individualized emails were sent to primary care clinicians participating in a VHA initiative (*n* = 43) inviting them to be interviewed. Individual semi-structured interviews were conducted September 2019 through January 2020, and were audio-recorded, transcribed, and analyzed using thematic analysis. The Consolidated Framework for Implementation Research (CFIR), a meta-theoretical framework of five domains associated with successful adoption of interventions, was used to organize findings.

**Results:**

Of 43 clinicians invited, 19 responded and were interviewed (44.2%). Findings represented two CFIR domains: Inner Setting and Characteristics of Individuals. For Inner Setting, three themes were identified as influencing implementation during the first year of the initiative. Clinicians reported a shared receptivity to implement buprenorphine, organizational support from pharmacy services and leadership, as well as cohesive relationships among implementation team members and collaboration with outside clinics. Noted barriers included fit within primary care workflows and lack of staff, time and access to onsite laboratory services and buprenorphine. For Characteristics of Individuals, two themes were identified that may facilitate clinicians’ willingness to provide buprenorphine care. Namely, clinicians reported positive attitudes about and experiences delivering opioid use disorder care and a willingness to learn/do something new.

**Conclusions:**

While implementation strategies should be tailored to individual clinic needs, prioritizing factors identified in this evaluation may support successful implementation of buprenorphine delivery in primary care.

**Supplementary Information:**

The online version contains supplementary material available at 10.1186/s13722-025-00568-9.

## Background

Opioid use disorder (OUD) poses a significant societal and public health challenge [1–4]. Recommended treatment includes medications for opioid use disorder (MOUD) [5, 6], which can reduce risk of opioid-related overdose and all-cause mortality [7–11]. Because of the number of opioid-related overdoses and the limited capacity of substance use treatment programs in the United States, expanding access to MOUD, specifically buprenorphine, in primary care is considered a critical response to the opioid overdose crisis. However, buprenorphine continues to be underutilized in primary care.

Although several factors have been reported as influential to the adoption of buprenorphine, most studies focused on buprenorphine in primary care have been based on the perspectives of clinicians who have limited experience with providing or trying to provide buprenorphine care [12–17]. Fewer studies have assessed factors associated with buprenorphine adoption in the context of national implementation initiatives [18–20]. Understanding the perspectives of providers in this context can provide valuable information about how barriers to and facilitators of adoption may be evolving as implementation interventions are rolled out.

The most frequently-cited barriers to buprenorphine adoption, such as waiver training requirements, concerns with being inundated by referrals, lack of time and lack of mental health, addiction, and behavioral health support, are based largely on expected rather than actual experiences [12–18]. Barriers to buprenorphine access reported in the literature include stigma, insurance limitations (e.g., prior authorizations) and pharmacy-level access challenges (e.g., limited buprenorphine supply), patient-level social determinants of health which create access and engagement barriers, and the challenge of addressing OUD in the setting of chronic pain [21–23]. These barriers may influence primary care clinicians’ perspectives on providing buprenorphine care. Additionally, much of the reporting on factors associated with buprenorphine adoption focuses on barriers, with little mention of factors that might facilitate buprenorphine prescribing in primary care. Overall, there is a lack of clarity regarding the barriers and facilitators that clinicians encounter when buprenorphine care has been implemented in primary care.

Consistent with national priorities, the Veterans Health Administration (VHA) sought to expand access to buprenorphine in primary care, pain and mental health clinics using a multifaceted implementation approach in 2018 [24, 25]. The purpose of this qualitative evaluation is to assess and describe primary care clinicians’ perspectives on delivering buprenorphine care during the first year of the initiative and to identify barriers and facilitators associated with implementation.

## Methods

### Setting

This evaluation is part of a mixed-methods quality improvement evaluation of the Stepped Care for Opioid Use Disorder Train-the-Trainer (SCOUTT) initiative, a national VHA effort to expand access to buprenorphine care in general healthcare clinics. This qualitative assessment was a partnership between the VHA Office of Mental Health and Suicide Prevention and an evaluation team. This evaluation was classified as quality improvement by the Institutional Review Board at Veterans Affairs (VA) Puget Sound and did not require human subjects’ approval. The Standards for Reporting Qualitative Research was used as a guideline to report findings [26]. 

The SCOUTT initiative has been previously described [24, 25]. Briefly, SCOUTT is an ongoing multi-site initiative, initiated in 2018 with a request for all 18 regional VHA networks (i.e., a network of facilities that are geographically interconnected and collaborate to provide healthcare services to veterans) to identify one facility and an interdisciplinary team of clinicians and clinical leaders to deliver buprenorphine care in at least one general healthcare clinic (i.e., primary care, pain, or general mental health). The interdisciplinary team of clinicians (or implementation team) was encouraged, but not required, to include an advocate (or clinical champion), a prescriber (i.e., physician, nurse practitioner or physician assistant), a registered nurse, a therapist (i.e., psychologist, social worker, or addiction therapist), and a clinical pharmacist. Each facility was also encouraged to assemble a team of clinicians specializing in the treatment of substance use disorder to consult with implementation teams. Implementation teams received training in buprenorphine care, stepped care, and models of care delivery at an in-person conference and were provided access to monthly education, external facilitation calls and resources from dedicated websites. As part of a larger evaluation of clinics participating in the initiative during the first year, this manuscript focuses on primary care clinics.

### Framework and interview guide

The semi-structured interview guide was developed using the Consolidated Framework for Implementation Research (CFIR), a meta-theoretical framework of factors that are associated with the adoption of interventions, that is often used to evaluate transformation initiatives, such as SCOUTT [27]. Questions were primarily designed to assess two of the five CFIR domains: inner setting and individual characteristics. Specifically, questions assessed experiences treating OUD with medications, how delivery of buprenorphine care fit with existing work processes/practices, and experiences working with other colleagues to treat patients with OUD (Supplemental Appendix).

### Participants and data collection procedures

We aimed to interview the clinical champion and at least one clinician from primary care implementation teams of 11 primary care clinics, using a snowball sampling approach [28]. Invitations were first sent to clinical champions and then to additional implementation team members who were identified by clinical champions. Implementation team members were sent up to three individualized emails inviting them to complete interviews.

Telephone interviews were conducted by two experienced qualitative interviewers beginning in September 2019, approximately one year after initiative launch, through January 2020. Participants were informed that the interviewers were part of the SCOUTT evaluation team, whose aim was to evaluate the implementation of the initiative. There were no direct relationships between the interviewers and participants. With consent, interviews were audio-recorded and transcribed. Two clinicians declined to be audio-recorded and detailed notes taken during the interviews were used as transcripts. Interviews lasted approximately 40 min. Interviewees completed a brief demographic and training history questionnaire.

### Data analysis

Interview transcripts were reviewed and collaboratively coded in ATLAS.ti by AL, AD, and DB using a combination of deductive and inductive thematic analysis, a flexible approach to thematically organize and analyze qualitative data [29]. Coders developed a codebook using a subset of transcripts and the CFIR framework [30]. The codebook was iteratively revised and modified when applied to the remaining transcripts, and previously coded interviews were reviewed following revisions. The coders met regularly to discuss codes and ensure consensus, with discrepancies resolved through discussion and data review. Thematic saturation was reached when no new information or themes were observed in these data [31]. The coded data were analyzed to identify and group principal concepts related to providing buprenorphine care, resulting in several themes and subthemes [30, 32]. 

## Results

Table 1 shows the demographic characteristics of clinicians interviewed. Of 43 clinicians invited, 19 were interviewed (44.2%), 1 declined and 23 did not respond. During the recruitment of interviewees, we learned that composition of team members varied across implementation teams, with clinical champions who were often physicians and pharmacists the most common members. Further, these two disciplines were the most engaged in buprenorphine care among team members. The average age was 42.4 (SD = 10.9), and the majority were women (52.6%), White (78.9%), and Medical Doctors or Doctors of Pharmacy (31.6% each). On average, clinicians had been practicing 14.0 (SD = 10.1) years overall and 9.2 (SD = 7.3) years at the VHA. Participants represented 8 VHA regional networks and 8 primary care clinics.

**Table 1:** Demographic characteristics of primary care clinicians interviewed

	(*n* = 19)	
**Characteristic**	**M or n**	**SD or %**
Age, Years, M(SD)	42.4	10.9
Gender		
Female	10	52.6
Male	9	47.4
Race		
Asian	2	10.5
Black or African American	2	10.5
White	15	78.9
Hispanic/Latino	1	5.3
Clinical Training		
Medical Doctor	6	31.6
Advanced Registered Nurse Practitioner	1	5.3
Doctor of Pharmacy	6	31.6
Registered Nurse	3	15.8
Doctor of Philosophy	1	5.3
Social Work	2	10.5
Years in Practice, M(SD)	14.0	10.1
Years at VA, M(SD)	9.2	7.3
Years in Clinic, M(SD)	5.4	5.8
Percent Patient Time, M(SD)	22.2	13.5

M = Mean

SD = Standard Deviation

VA = Department of Veterans Affairs

We report on findings from two CFIR domains that are relevant to developing strategies for implementation of MOUD in primary care: Inner Setting and Characteristics of Individuals. Three themes were identified within the Inner Setting domain as influencing implementation during the first year of SCOUTT: Shared Receptivity of Individuals to Implement Buprenorphine, Organizational Support of Buprenorphine Implementation, and Networks and Communication. Two themes were identified within the domain Characteristics of Individuals related to clinicians’ willingness to deliver buprenorphine: Clinicians’ Knowledge and Beliefs about OUD Treatment and Personal Attributes. To define level of endorsement, ‘most’ is defined as > 50% of respondents (*n* = 19), ‘many’ as 20–50%, and ‘few’ as < 20%.

### CFIR domain: inner setting

#### Shared receptivity of individuals to implement buprenorphine

This theme represents the degree to which clinicians held perceptions in common that influenced MOUD implementation in their clinics. Two subthemes were identified that reflected shared perceptions about the relative priority or advantage of buprenorphine access in primary care and the fit of OUD treatment in clinic workflows.

### Relative priority/advantage

Most described buprenorphine as an important service for primary care patients, highlighting the value of interventions that can be delivered in primary care to reduce the risk of opioid overdose and help stabilize and positively change their patients’ lives.

Clinician1: I’m a firm believer in expanding access to [MOUD]. I think we have a lot of opportunity to help our patients.... I hope we get more momentum and we’re able to get more providers onboard to further expand access throughout the healthcare system.

Clinician2: It’s a fantastic tool, especially if you’re dealing with patients who are high risk, just mitigating that risk.... So just being able to have Suboxone as an option and seeing people do well with it has definitely been positive.

### Clinic fit

Overall, perspectives were mixed with many noting that OUD treatment fits within primary care workflows, while many reported that it does not. Clinicians who reported that buprenorphine care is consistent with primary care workflows highlighted the mission of their primary care clinic and resources (e.g., capacity, staff, time, space), supporting implementation.

Clinician3: I think it fits really well, especially with our clinic because our Primary Care Clinic is specifically focused on Opioid Use Disorder and chronic pain. And I think SCOUTT has really prepared our team to serve the veterans at a higher level than if we were not a part of the program. A lot of different disciplines have been educated on the same material, which is great.

However, the additional time and visits needed to initiate and provide follow-up buprenorphine care while simultaneously meeting the VHA priority to increase primary care access for all patients were the most common reasons given for incompatibility.

Clinician4: So I would say not well because currently the need for induction is problematic.... [I] haven’t done it myself, and my nurses haven’t done it, and my clinic hasn’t done it, it’s so far not compatible because it’s not part of the daily business, right?

### Organizational support of buprenorphine implementation

This theme reflects indicators of organizational commitment to MOUD implementation in primary care, including specific resources and engagement of leadership in support of implementation. Key subthemes representing facilitators of buprenorphine implementation included support of pharmacy services and engagement of leadership, while barriers included lack of staff, time and onsite access to laboratory services and buprenorphine.

### Pharmacy services

Many reported positive interactions with pharmacy services at their facility, highlighting general support and strong collaboration with clinical pharmacists in direct delivery of buprenorphine care.

Clinician5: And then we worked closely with Pharmacy, with getting them to know the normal processes as to when we induce, to have like a process already there.... Pharmacy has been really great about that, having access to all of that communication.

Additionally, most spoke to the collaborative care arrangement with pharmacists, noting their direct role in providing multiple aspects of buprenorphine care, including identifying patients who need care, providing education to patients and providers, outreaching, scheduling/coordinating care, and delivering medication management services.

Clinician6: And then we do a co-management approach, so basically a collaborative care team. I work really close with the PACT Pharmacist. I’ll see the patient at least once and get the buprenorphine started. And then the pharmacist will follow up with them pretty frequently at first. And then we decrease that over time.

### Leadership

Most described leadership involvement as a facilitator, noting a general level of support or buy-in for improving access to buprenorphine, attendance at meetings (e.g., SCOUTT conference, local team meetings), and resources (e.g., time to get waivered, staffing, decreased panel size).

Clinician7: I think we’re fortunate to have a leadership team who was really supportive of expanding the treatment of OUD within the Primary Care setting.... [T]hey’ve also been supportive of things to help facilitate that, things like decreasing providers’ panel sizes...

### Staff and time

Many reported staffing barriers, noting loss of staff who were originally part of SCOUTT and a need for additional staff (e.g., a project manager, administrative support, nursing). Many identified time as a barrier, citing a lack of availability for follow-up appointments and limited time to focus on the implementation initiative because of a national VHA priority to increase primary care access.

Clinician8: Time has been a concern, because as we know, Primary Care is always under pressure... [W]e understand destigmatizing [OUD treatment] and making it something that a patient can just discuss regularly at a visit, but how do we incorporate that in? I would say there’s some adjustments for us as far as time management. I wouldn’t label it impossible..., but that initial phase...was a little bit more difficult because you’re in that development phase.

### Lab and medication access

Some primary care clinicians not located at the larger medical centers with comprehensive services reported a lack of onsite laboratory services (e.g., urine drug screens) and access to buprenorphine. Clinicians at these clinics reported needing to send laboratory orders to other facilities for processing or requiring patients to have laboratory work performed at other facilities prior to their appointment. Further, they reported a lack of access to buprenorphine because a pharmacy was not onsite, sharing that patients needed to go to other facilities or receive buprenorphine via the mail– steps that potentially delayed or complicated buprenorphine initiations.

Clinician9: I think more difficult to treat within Primary Care due to not having the lab or pharmacy on site. And, not being successful at navigating a mechanism for point of care testing.

### Networks and communication

This theme represents the strength and quality of informal and formal connections and communications within teams, and with other colleagues and other services. Key subthemes that positively influence implementation of buprenorphine include how teams function and work together and their relationships with other colleagues and/or clinics.

### Team functioning

Most described the importance of a team-based approach to patient care (including different roles treating patients simultaneously), cohesive relationships among team members, respect for team members’ roles and interdisciplinary expertise, and frequent communication (e.g., interdisciplinary meetings to coordinate care).

Clinician5: I work with a [physician] and a[nurse practitioner]... [W]e have a team psychologist, physical therapist, pharmacist, a part time social worker. And we have interdisciplinary meetings for these patients... But prior to the initial visit, myself and the social worker, we do an intake for the patient where we do the personal health inventory... So once I’ve done my assessments, I pass it on to them prior to, and have a small discussion before they actually see the patients. So actually, the patient’s initial visit is done by the provider and the psychologist and physical therapist, they have an hour-long visit, and then that patient’s case is again discussed... with the interdisciplinary team in a meeting. So everybody has touched that patient, the whole team, in one way or another.

### Relationships with other clinics/colleagues

In addition to within clinic collaboration, many reported collaborating with and outreaching to other clinics (e.g., mental health, pain, other primary care clinics) to develop relationships, regular communication, consultation, collaboration on clinic policy (e.g., patient flow), and easy referrals/patient sharing.

Clinician2:...if I were to have concerns that they need more resources than we can offer in our clinic, we work closely with our [substance use disorder (SUD) treatment providers], and we’ll pass patients back and forth if needed. And we have just great support with the Mental Health Team, so I guess any concerns are well addressed in terms of collaborating with our colleagues.

With respect to their facility’s SUD treatment providers, most reported that these colleagues were easy to reach, amenable to being shadowed, provided consultation around patient care questions, and had open referral pathways for patients to step up or step down in level of care.

“Cinician1: Having support from our local Addiction Treatment Services. So making sure that they were involved from the beginning, and meeting with them regularly, and working with them together on this initiative, I think that’s been very important... [I]t’s very important for us to have support and to discuss cases with them, so we felt more comfortable managing them at our level. And trying to follow stepped care, that’s a vital connection there from Addiction Treatment Services to something like Primary Care... Just having them available, quickly available through messaging or phone calls, and regular meetings, has been very helpful.”

### CFIR domain: characteristics of individuals

#### Clinicians’ knowledge and beliefs about OUD treatment

This theme represents clinicians’ beliefs about OUD treatment that may facilitate provision of buprenorphine in primary care. Two subthemes were identified that reflect clinicians’ knowledge and beliefs about treatment of OUD compared to other chronic conditions and clinicians’ reports on patient improvement and clinician treatment experiences.

### OUD treatment compared to treatment of other chronic conditions

Most reported few differences between treatment of OUD relative to treatment and follow up of other chronic health conditions such as diabetes and heart disease.

Clinician10: I really don’t think there are many differences at all.

However, clinicians did note various features of OUD care they considered somewhat different, though not necessarily barriers to OUD treatment. For example, clinicians noted greater regulatory scrutiny as well as a need for psychosocial support and more frequent follow-up visits for OUD relative to other chronic health conditions.

### Patient improvement and clinician treatment experiences

Many reported seeing clinically and functionally meaningful progress in patients’ symptoms and lives since initiating buprenorphine, highlighting how helpful buprenorphine can be and how quickly improvements appeared to happen, and described providing buprenorphine care as rewarding and satisfying. Further, clinicians reported that treating OUD facilitated treatment of other medical comorbidities and/or chronic conditions and improves patients’ overall well-being and health (e.g., improving self-care, physical health and housing).

Clinician11: In Primary Care we are incrementalists and often think of things over a very long term, but this is one of the few medications I can provide where I can see a very quick and drastic change in lives... [Y]ou quickly see individuals who, within a matter of months, have gone back to school or are more active in their family’s lives, or are feeling healthier and better about themselves, have changed in terms of their mood, in terms of their depression, happiness and outlook. So, in terms of satisfaction, rare is the intervention in Primary Care where you see that turnaround in a short period.

Clinician6: It’s an effective treatment that helps people get better, and it’s really fun to see the patients. And I think that’s important for people to hear, that this is fun. And people do well. Not everybody, but some of them do great. And they’re really grateful.

### Personal attributes

This theme reflects clinicians’ reasons for providing buprenorphine care. Many reported a willingness to learn and do something new, to be flexible and resilient, and to advocate for increasing access to buprenorphine care.

Clinician12:... I read all kinds of stuff on it, I tried to work with our Suboxone nurses to see what they do in their clinics, I got information and pamphlets I could give the veterans on Suboxone, so that they had all of the information. I learned all about the COWS and how to ask all of those questions. I got the template, or the consent.

## Discussion

This evaluation identified key factors in the Inner Setting and Characteristics of Individuals CFIR domains related to the adoption of OUD care in VHA primary care settings from the perspective of clinicians delivering care as part of a national implementation initiative. Given that the existing literature focuses largely on clinicians who have little experience treating or trying to treat OUD in primary care, such perspectives are critical. Inner Setting themes that emerged as supporting implementation during the first year of the initiative included a shared receptivity to OUD care delivery, cohesive relationships among implementation team members, organizational support through pharmacy services and leadership, and collaboration with outside clinics. While facilitation supports and resources were in place through the SCOUTT initiative, clinicians struggled with barriers identified in existing literature, including fitting buprenorphine care within primary care workflows, lack of staff and time and access to onsite laboratory services and buprenorphine. Key Characteristics of Individuals that appeared to influence adoption included individual clinicians’ attitudes about and experiences delivering OUD care, as well as personal attributes.

Functional interdisciplinary teams with a shared vision were identified as a facilitator to implementation [19]. Clinicians reported cohesive relationships among team members in which the role of each member is valued as central to their efforts. Respect for team members’ roles and expertise, in addition to frequent communication (particularly to coordinate patient care), offer practical guidance to foster positive team dynamics and build team cohesion. Although cohesive relationships among teams is highlighted as an important facilitator, the majority of interviewees were clinical champions, often physicians, and pharmacists. Thus, it is not clear if cohesiveness among all team members is important or only among team members most engaged in delivering buprenorphine care. Some teams relied primarily on physicians and pharmacists to provide buprenorphine care. Pharmacists are trained to provide comprehensive medication management, using in-depth knowledge of medications and disease states to manage medication therapy, and thus are well-positioned to deliver buprenorphine care for OUD. Collaboration between only physicians and community pharmacists is a model that has been shown to be feasible and effective for the management of opioid use disorder in prior studies, suggesting that collaboration among several interdisciplinary team members may not be necessary [33]. A model that relies on fewer members to deliver care may hold greater promise in less integrated healthcare systems or those with fewer resources.

In addition, most clinicians reported positive and collaborative relationships with clinics outside their own, particularly with their facility’s SUD specialty care program [12, 13, 15, 17]. Key attributes of SUD clinics were accessibility, consultation, and referral pathways to step up or down in level of care. It is important to note that SUD specialists were purposefully included in the SCOUTT initiative and that VHA is an integrated healthcare system, allowing for the development of working relationships and clear referral pathways. This type of collaboration may not be as feasible in less integrated healthcare systems. However, these findings highlight the importance of building such relationships with local SUD specialty care resources and are a noteworthy consideration when developing an implementation strategy.

Regarding organizational support, pharmacists were identified as key team members. While not originally a targeted SCOUTT model, a collaborative care model between a physician and a pharmacist emerged as a viable option to providing buprenorphine care in VHA primary care, which may be less feasible in non-VHA settings. Pharmacists provide comprehensive patient care and medication management and are well suited to having a primary role in OUD care [34]. Additionally, physician-pharmacist relationships appeared to reduce some concerns related to limited time, which is consistent with emerging research [33]. Future research should continue to examine the role of pharmacists in a collaborative care model, particularly related to overcoming logistical concerns.

Consistent with prior research, logistical concerns such as staff and time constraints were reported [12, 14–19, 35]. Further, results were mixed regarding buprenorphine care fitting within primary care workflows, and limited access to onsite laboratory services and buprenorphine were barriers for some clinics not located at the larger medical centers with comprehensive services. Of note, despite clinicians’ receptivity to providing OUD care, these barriers were still present even with the facilitation effort of SCOUTT, and therefore may be especially important considerations for primary care clinics wishing to implement MOUD. Future research to evaluate whether these concerns dissipate over time may be warranted.

Most primary care clinicians viewed OUD care as comparable to the treatment of other chronic medical conditions and were motivated to provide care. A positive attitude toward buprenorphine as a treatment option and a sense of responsibility to treat OUD in primary care have been reported as facilitators [12, 14, 17–19]. Notably, clinicians described seeing improvements in patients’ lives as satisfying and motivating [35]. Many reported that providing buprenorphine care helped their patients to stabilize quickly, which allowed them to address other medical needs (e.g., other chronic disorders, improvements in physical health). Thus, the provision of OUD care in and of itself may be a facilitator to adoption. Having colleagues who advocate for providing OUD care, based on direct experiences combined with the viewpoint that it is a rewarding aspect of their practice, may serve as a catalyst for others to provide this care. Fostering opportunities for clinicians to share personal experiences with their colleagues via case presentations and local workshops may be a strategy worth formalizing. Future research on patient satisfaction and health outcomes, as well as evaluating whether the factors identified in this evaluation help sustain the provision of buprenorphine care into practice within primary care, is warranted.

This evaluation has limitations. SCOUTT provided on-going consultation and resources which likely influenced the results. Barriers to implementation may be more prominent for clinics and clinicians not participating in this initiative. Generalizability is limited to VHA clinicians who agreed to be interviewed. Thus, these findings may reflect the experiences and opinions of clinicians who may have been more engaged with the initiative and supportive of OUD care. As clinical champions were most often physicians and pharmacists, nearly two-thirds of the respondents interviewed were physicians or pharmacists, limiting representation from other disciplines and the possibility of comparisons between disciplines. Future evaluations of the SCOUTT initiative should assess whether factors associated with implementation vary by discipline. Further, these findings may not generalize to non-VHA primary care clinics, especially those that are not part of a healthcare system with integrated services (e.g., pharmacy, mental health, specialty care) and resources to address social determinants of health (e.g., housing instability) often needed by persons affected by substance use, or to non-veteran populations. Further, these findings predate the emergent trend in fentanyl use, which has made effective control of withdrawal and adherence to buprenorphine treatment more challenging [36, 37], the removal of the x-waiver requirement for buprenorphine prescribing, often-reported as a barrier to buprenorphine adoption, and changes in practices as a result of the COVID-19 pandemic, such as the increase in virtually-delivered buprenorphine care and reduction in use of urine drug screens. Results do not indicate the size or impact of a particular factor, its impact on treatment decisions, or how factors impact one another. We also did not evaluate whether factors vary by clinician characteristics, clinic settings or by the degree to which buprenorphine care was implemented in a clinic, factors that are important to assess in future evaluations of the SCOUTT initiative. It is important to note that the SCOUTT initiative is an ongoing effort, with both original implementation clinics and new implementation clinics continuing to participate in improving access to buprenorphine care allowing for future research on how buprenorphine care evolves over time.

## Conclusion

This evaluation strengthens our understanding of the implementation of OUD care in primary care clinics participating in a national implementation initiative. Given the need for expanded access to OUD care, identifying the facilitators as well as the barriers to implementation is critical. While implementation strategies should be tailored to individual clinic needs, prioritizing the inner setting and individual characteristics factors identified in this evaluation may facilitate successful implementation of buprenorphine care in other healthcare settings.

## Electronic supplementary material

Below is the link to the electronic supplementary material.

Supplementary Material 1

Supplementary Material 2

## Data Availability

The datasets generated and analyzed during the current evaluation are available from the corresponding author on reasonable request.

## Abbreviations

CFIR: Consolidated Framework for Implementation Research
MOUD: Medications for opioid use disorder
OUD: Opioid use disorder
SCOUTT: Stepped Care for Opioid Use Disorder Train-the-Trainer
VA: Veterans Affairs
VHA: Veterans Health Administration
