# Supplementary material for: Implementing buprenorphine for opioid use disorder in veterans health administration primary care: a qualitative analysis
Source: Addict Sci Clin Pract. 2025 Apr 30;20:38. doi: 10.1186/s13722-025-00568-9 (PMC12042572; doi:10.1186/s13722-025-00568-9)
Supplement: Supplementary file 1 — Supplementary Material 1 [file 13722_2025_568_MOESM1_ESM.docx]

**Supplemental Appendix:** Stepped Care for Opioid Use Disorder Train-the-Trainer (SCOUTT) Interview Guide

**Interviewer Name:**

**Subject ID:**

**Date:**

**Time Start:**

**Time End:**

Hello [Dr./Mr./Ms. ­­­­______],

Thank you for your participation. I would like to ask you questions about your experiences treating patients with opioid use disorder.

This interview will take approximately 20-45 minutes to complete. Please know that you can stop the interview at any time, and are free to skip any questions that you would prefer not to answer.

Because we want to keep responses confidential, we ask that you do not use any names during this interview.

Do you have any questions?

To capture the information you give us, we would like to record this interview.  Is this okay with you?  [Hit record button.]  Okay, to confirm, I’m starting the recording.  Is this ok with you?

| **Grounded Probes:** *If responses to any of the questions are limited or require clarification, probes may be used to illicit more detailed responses. Probes should use words or phrases presented by the participant using one of the following formats:*  a. What do you mean by ____________?  b. Tell me more about ____________.  c. Give me an example of ____________.  d. Tell me about a time when ____________.  e. Is there anything that makes ____________ easier?  d. Is there anything that makes ____________ difficult? | | |
| --- | --- | --- |
| **CFIR Domain(s)** | **Question** | **Probe** |
| **Characteristics of Individuals** | What is your role on the SCOUTT team? | Describe how SCOUTT has impacted your job/role? |
| **Intervention Characteristics**  **Characteristics of Individuals** | Describe your experiences treating opioid use disorder with medications in clinic? | What concerns do you have, if any, about treating opioid use disorder?  How has prescribing medications to treat opioid use disorder helped you meet the needs of your patients? |
| **Outer Setting** | Describe how you identify patients who need to be assessed for an opioid use disorder? |  |
| **Intervention Characteristics**  **Characteristics of Individuals** | What are the differences, if any, of treating other chronic disorders versus treating opioid use disorder? | How straight forward or easy is treating opioid use disorder? |
| **Inner Setting** | How well does treating opioid use disorder with medications fit with existing work processes and  practices? |  |
| **Inner Setting** | Describe how you work with other colleagues in clinic to treat patients with opioid use disorder? |  |
| **Inner Setting** | To what extent do you feel like you are expected to treat opioid use disorder? | How much pressure do you feel to implement treatment for opioid use disorder? (From whom?) |
| **Inner Setting** | What tools, resources and support did you need to begin treating opioid use disorder in your clinic? | Do you have remaining resource needs? |
| **Intervention Characteristics** | Describe how helpful, or not helpful, the SCOUTT training (held in August 2018) was? | Who can you contact for advice if you have questions about how to treat a patient?  Do you have remaining training needs? |
|  | Describe your experiences with SCOUTT facilitation activities? (For example.: monthly calls, SP site, site visits, working with the facilitation team)? |  |
| **Spread (Not A CFIR Domain)** | What has been your experience trying to train another clinic/provider in your facility? |  |
| **Intervention Characteristics**  **Inner Setting**  **Characteristics of Individuals** | Are there any other factors that we haven’t discussed yet that made it easy or difficult to treat opioid use disorder in your clinic? |  |
